# Designed hybrid nanostructure with catalytic effect: beyond the theoretical capacity of SnO_2_ anode material for lithium ion batteries

**DOI:** 10.1038/srep09164

**Published:** 2015-03-17

**Authors:** Ye Wang, Zhi Xiang Huang, Yumeng Shi, Jen It Wong, Meng Ding, Hui Ying Yang

**Affiliations:** 1 Pillar of Engineering Product Development, Singapore University of Technology and Design, 8 Somapah Road, 487372, Singapore

## Abstract

Transition metal cobalt (Co) nanoparticle was designed as catalyst to promote the conversion reaction of Sn to SnO_2_ during the delithiation process which is deemed as an irreversible reaction. The designed nanocomposite, named as SnO_2_/Co_3_O_4_/reduced-graphene-oxide (rGO), was synthesized by a simple two-step method composed of hydrothermal (1^st^ step) and solvothermal (2^nd^ step) synthesis processes. Compared to the pristine SnO_2_/rGO and SnO_2_/Co_3_O_4_ electrodes, SnO_2_/Co_3_O_4_/rGO nanocomposites exhibit significantly enhanced electrochemical performance as the anode material of lithium-ion batteries (LIBs). The SnO_2_/Co_3_O_4_/rGO nanocomposites can deliver high specific capacities of 1038 and 712 mAh g^−1^ at the current densities of 100 and 1000 mA g^−1^, respectively. In addition, the SnO_2_/Co_3_O_4_/rGO nanocomposites also exhibit 641 mAh g^−1^ at a high current density of 1000 mA g^−1^ after 900 cycles, indicating an ultra-long cycling stability under high current density. Through *ex-situ* TEM analysis, the excellent electrochemical performance was attributed to the catalytic effect of Co nanoparticles to promote the conversion of Sn to SnO_2_ and the decomposition of Li_2_O during the delithiation process. Based on the results, herein we propose a new method in employing the catalyst to increase the capacity of alloying-dealloying type anode material to beyond its theoretical value and enhance the electrochemical performance.

Tin oxide (SnO_2_) is deemed as one of the most promising anode materials of lithium ion batteries (LIBs) to replace the commercial graphite anode due to its high theoretical capacity (~782 mAh g^−1^), high abundance, low cost and relative low charge-discharge plateau compared to other metal oxides^1^,^2^,^3^. However, SnO_2_ anodes suffer from three main limitations: (i) more than 200% volume change during the lithiation and delithiation process, resulting in severe electrode pulverization and fast capacity fading during the cycle process; (ii) low electrical conductivity reducing the electron transport, leading to relatively low rate capability; (iii) poor initial Coulombic efficiency (CE) due to the irreversible conversion reaction during the initial lithiation process, resulting in the additional cathode material consumption^4^,^5^,^6^. Many research efforts have been devoted in solving issues (i) and (ii) through the use of unique morphological design and incorporation of carbon based materials^7^,^8^,^9^,^10^,^11^,^12^,^13^,^14^,^15^,^16^,^17^,^18^. Various morphologies including zero dimensional (0D) nanoparticles, one dimensional (1D) nanorods/nanowires/nanotubes, two dimensional (2D) nanosheets and three dimensional (3D) nano-hollow structures have been explored as different morphologies may affect the surface area and lithium ion kinetics^10^,^11^,^15^,^19^,^20^,^21^. For example, Wang et al. designed SnO_2_ nanoboxes which can deliver a capacity of 570 mAh g^−1^ at 0.2 C even after 40 cycles^22^. On the other hand, carbonaceous materials, which are ideal volume change buffers and good electron conductors, have been widely employed to enhance the electrochemical performance of SnO_2_ nanocomposites. SnO_2_/graphene nanosheets (SnO_2_/GNS) nanostructure synthesized by Paek et al. achieved a reversible capacity of 810 mAh g^−1^ 13. The improved performance was attributed to the reduced volume change and increased conductivity with the assistance of graphene nanosheets^13^. In addition, graphene nanosheet was also found to be useful in preventing the aggregation of SnO_2_ nanoparticles during the lithiation and delithiation process^23^.

However, issue (iii) is seldom solved due to the inherent irreversible conversion reaction of SnO_2_ to Sn (equation 1) which is followed by the subsequent alloying reaction (equation 2, forward process) during the initial lithiation process^11^,^15^:
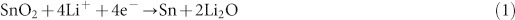

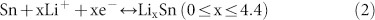
In equation 1, 1 mole of SnO_2_ is reacted with 4 moles of Li-ions to form 1 mole of Sn and 2 moles of Li_2_O. Subsequently, 1 mole of Sn atom is able to form Li_x_Sn alloy with a maximum of 4.4 moles of Li-ions during the alloying process shown in equation 2. In other words, as much as total 8.4 Li-ions can be utilized during the first lithiation process. However, due to irreversibility of the reaction in equation 1, at least 4 moles Li-ions become inactivated in the following delithiation/lithiation cycles, leading to a low initial CE (~40%)^15^. Therefore, if the conversion reaction of equation 1 is reversible, the theoretical specific capacity of SnO_2_ can be increased from 782 to 1493 mAh g^−1^ (based on 8.4 Li ions), which is far beyond the theoretical capacity of SnO_2_.

Recently, SnO_2_ anodes with higher specific capacity than the theoretical value have also been reported, the increased capacity was mainly attributed to partial reversible conversion of Sn to SnO_2_ coupled with a synergistic effect with carbonaceous material or other nanoparticles^24^,^25^. Chen et al. reported SnO_2_-SiC coated by graphene layer core-shell structure can deliver a reversible capacity of 810 mAh g^−1^ and retain ~83% initial capacity after 150 cycles^26^. The improved performance was attributed to the reversible conversion reaction of Sn to SnO_2_^26^. However, up to now the detailed mechanism of this reversible conversion reaction still remains unclear. In a similar case, it has been reported that transition metal particles can play a critical role for the reoxidation of metallic germanium during its delithiation process for the MGeO_3_ (M = Cu, Fe and Co) nanocomposites^27^,^28^. This phenomena was attributed to the presence of metallic nanoparticles which are able to promote the decomposition of Li_2_O and also form a conductive network to accelerate the reoxidation of Ge^28^. Therefore, we believe that transition metal nanoparticles can also be used to enhance the electrochemical performance of SnO_2_ nanocomposites.

Herein, Cobalt (Co) nanoparticle catalyst is used to promote the reoxidization of Sn to SnO_2_ and the decomposition of Li_2_O during the delithiation process. Since nano-sized Co is easily oxidized, Co_3_O_4_ nanoparticle is synthesized in the nanocomposites instead of Co. At the same time, reduced graphene oxide (rGO) is used in the nanocomposites system to increase the conductivity and reduce the volume change during the lithiation/delithiation process. The designed SnO_2_/Co_3_O_4_/rGO nanocomposites exhibit an excellent electrochemical performance, in terms of large specific capacity, high rate capability and long cycling stability. The detailed mechanism is also proposed based on detailed *ex-situ* transmission electron microscopy (TEM) investigations.

## Results

**Figure 1:**
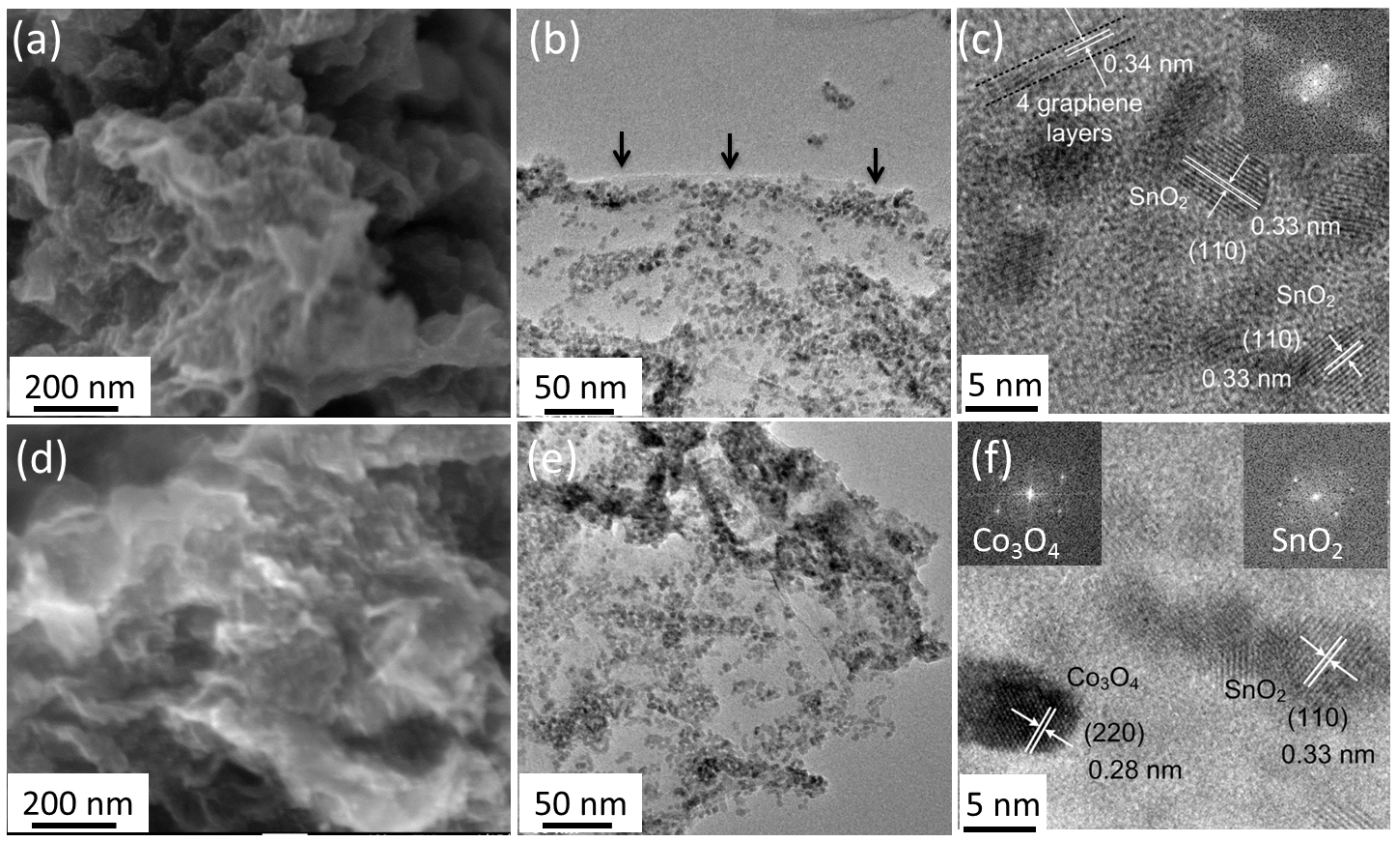
Morphology of SnO_2_/rGO and SnO_2_/Co_3_O_4_/rGO hybrid nanocomposites. (a) SEM, (b) low- and (c) high-resolution TEM images of SnO_2_/rGO nanocomposites. Inset in (c) is the SAED pattern of SnO_2_ nanoparticles of SnO_2_/rGO nanocomposites. (d) SEM, (e) low- and (f) high-resolution TEM images of SnO_2_/Co_3_O_4_/rGO nanocomposites. Inset in (f) is the SAED patterns of Co_3_O_4_ and SnO_2_ nanoparticles of SnO_2_/Co_3_O4/rGO nanocomposites.

The morphology of the SnO_2_/rGO and SnO_2_/Co_3_O_4_/rGO nanocomposites is shown in Figure 1. GO was reduced into rGO after the synthesis of SnO_2_ nanoparticles by thermal reduction and reductant (NH_4_H_2_PO_4_)^29^,^30^. The synthesized nanocomposites exhibit typical morphology of metal oxide nanoparticles grown on folded rGO sheet (Figure 1a)^31^. It is widely accepted, and evident in these images, that the usage of rGO can effectively prevent the aggregation of metal oxide nanoparticles which often results in the peeling off from the current collector. Moreover, the folded rGO creates a porous structure which facilitates the penetration of electrolyte into the hybrid structure to enhance Li ions accessibility. In order to show detailed structure morphology, TEM characterization was carried out and the images are shown in Figure 1b and 1c. It is clearly observed that the edge of rGO sheet (indicated by black arrows) and SnO_2_ nanoparticles, with the diameter ranging from 5 to 10 nm, are uniformly anchored on the rGO sheet as indicated in Figure 1b. There are 4 graphene layers (between two black dot lines) with interplanar spaces of 0.34 nm for graphene sheet as shown in Figure 1c. The interplanar spaces of the nanoparticles are 0.33 and 0.27 nm, corresponding to the (110) and (101) crystal planes of the tetragonal rutile-like SnO_2_, respectively, as shown by the high-resolution TEM (HRTEM) image of Figure 1c^26^. The diffraction dots in the selected area electron diffraction (SAED) pattern (insert in Figure 1c) indicate that the nanoparticle is nanocrystalline. The morphology of SnO_2_/Co_3_O_4_/rGO nanocomposites is shown in Figure 1d to 1f. It is apparent that the overall morphology of SnO_2_/Co_3_O_4_/rGO is almost the same as that of SnO_2_/rGO (Figure 1d) where the metal oxide nanoparticles are also uniformly grown on top of rGO sheet (Figure 1e). In order to distinguish SnO_2_ and Co_3_O_4_ nanoparticles, HRTEM was employed to identify the detailed structure as shown in Figure 1f. The interplanar spaces of the nanoparticles sheets are 0.33 and 0.28 nm, corresponding to the (110) crystal planes of the tetragonal rutile-like SnO_2_ and (220) crystal planes of Co_3_O_4_, respectively^32^,^33^. Moreover, energy dispersive x-ray (EDX) analysis was carried out to investigate various elements distribution shown in Figure S1 (see Supporting Information). Four elements of Sn, Co, C, and O are uniformly distributed in the nanocomposites, indicating that SnO_2_ and Co_3_O_4_ nanoparticles were homogeneously grown on rGO nanosheets. The control sample of SnO_2_/Co_3_O_4_ nanocomposites without rGO sheet was also prepared at the same time (see Supporting Information, Figure S2). The SEM image shows that SnO_2_/Co_3_O_4_ nanoparticles with an average diameter of 5–10 nm aggregate together forming a large cluster without rGO sheets. Information from the elements distribution from the EDX analysis in Figure S2b shows that two metal nanoparticles are uniformly distributed in the composites.

**Figure 2:**
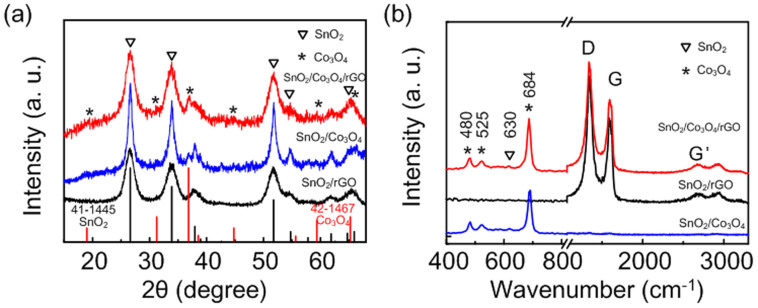
(a) XRD patterns and (b) Raman curves of SnO_2_/rGO and SnO_2_/Co_3_O_4_/rGO hybrid nanocomposites. The XRD peaks of SnO_2_ (indicated by triangles) in all samples are indexed to the tetragonal crystal structure of SnO_2_ (JCPDS No. 41-1445, black line in (a)) and the Co_3_O_4_ peaks (indicated by stars) in the samples are indexed to pure face centered cubic (fcc) crystal structure of Co_3_O_4_ (JCPDS No. 42-1467, red line in (a)).

The crystal structure of SnO_2_/rGO, SnO_2_/Co_3_O_4_/rGO and SnO_2_/Co_3_O_4_ nanocomposites was examined by X-ray diffraction (XRD) patterns, as shown in Figure 2a. The common peaks of the three samples, located at 26.6°, 33.9° and 51.8°, can be indexed as the (110), (101) and (211) planes of the tetragonal structure of SnO_2_ (JCPDS No. 41-1445) respectively^14^. The small peaks (indicated by stars) found in SnO_2_/Co_3_O_4_/rGO and SnO_2_/Co_3_O_4_ nanocomposites can be attributed to the pure face centered cubic (fcc) crystal structure of Co_3_O_4_ (JCPDS No. 42-1467)^34^. As Co nanoparticles only serve as catalyst in the nanocomposites, they were prepared with a low loading content of Co_3_O_4_ as compared to SnO_2_, leading to a weaker intensity for peaks corresponding to Co_3_O_4_. Raman spectra of SnO_2_/rGO, SnO_2_/Co_3_O_4_/rGO and SnO_2_/Co_3_O_4_ nanocomposites are shown in Figure 2b. The peaks located at 480, 525 and 684 cm^−1^ in SnO_2_/Co_3_O_4_/rGO and SnO_2_/Co_3_O_4_ nanocomposites are typical Raman peaks of Co_3_O_4_^35^. The very weak peak located at 630 cm^−1^ for SnO_2_/Co_3_O_4_/rGO sample is attributed *A*_1g_ vibration mode of SnO_2_^36^. On the other hand, the peaks located at 1384 and 1592 cm^−1^ for the SnO_2_/rGO, SnO_2_/Co_3_O_4_/rGO nanocomposites are attributed to the D- and G-peaks corresponding to the defects and disorder in the graphite layer, and the vibration of sp^2^-bonded carbon atoms in the hexagonal graphitic lattice, respectively^37^,^38^,^39^,^40^. The surface area of the pristine GO, SnO_2_/Co_3_O_4_ and SnO_2_/Co_3_O_4_/rGO were also performed by Brunauer–Emmett–Teller (BET) analysis shown in Figure S3 (see Supporting Information). The surface area of the synthesized SnO_2_/Co_3_O_4_/rGO (i.e., 244.5 m^2^ g^−1^) is more than three times higher than that of pure SnO_2_/Co_3_O_4_ (i.e., 60.9 m^2^ g^−1^) and GO (i.e., 76.8 m^2^ g^−1^), indicating that GO and metal oxides can effectively to prevent the restacking and agglomeration between each other resulting in greatly improving the surface area of SnO_2_/Co_3_O_4_/rGO nanocomposites. The same phenomenon has been observed in metal oxides with rGO nanocomposites^31^.

The content of metal oxide (SnO_2_, or SnO_2_/Co_3_O_4_) in the synthesized nanocomposites was measured by thermal gravimetric analyses (TGA) measurements conducted under air atmosphere and the corresponding curves are shown in Figure S4 (see Supporting Information). When the temperature is less than 100°C, a small weight 8% loss is observed due to the evaporation of moisture adsorbed on the surface of nanocomposites. At higher temperature, a further weight loss of 16% between 100 and 520°C is attributed to the oxidation of functional groups on rGO sheets and carbon in air^41^,^42^. Therefore, SnO_2_/rGO nanocomposites contain 82.6 wt% of SnO_2_ and 17.4 wt% of rGO. On the other hand, SnO_2_/Co_3_O_4_/rGO nanocomposites contain 83.6 wt% of SnO_2_/Co_3_O_4_ and 15 wt% of rGO. Therefore, the SnO_2_/Co_3_O_4_/rGO nanocomposites can be expressed as SnO_2_(Co_3_O_4_)_0.174_/rGO (see Supporting Information for detail calculation of Co_3_O_4_ content in the nanocomposites in the explanation of Figure S4). It is worth mentioning that the temperature for the weight loss owing to the oxidization of rGO for SnO_2_/Co_3_O_4_/rGO nanocomposites is lower than 380°C, which is much lower than that of SnO_2_/rGO nanocomposites. The possible reason may be attributed to the catalytic role of Co_3_O_4_ nanoparticles to reduce the oxidation temperature of rGO. The same phenomenon was also reported in Co_3_O_4_/GO and Co_3_O_4_/CNT hybrid nanostructure^43^,^44^.

**Figure 3:**
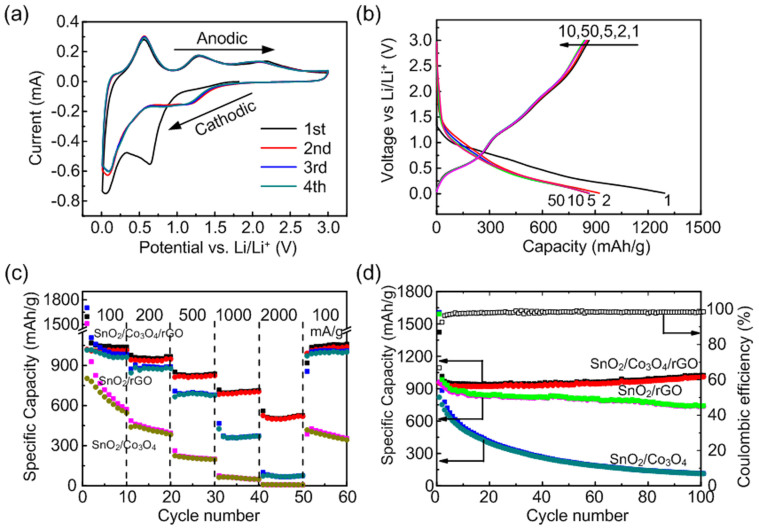
(a) CV curves of SnO_2_/Co_3_O_4_/rGO nanocomposites electrode of the first 4 cycles at a scan rate of 0.1 mV s^−1^ in a potential range of 0.01–3 V *vs.* Li/Li^+^. (b) Galvanostatic discharge/charge curves of SnO_2_/Co_3_O_4_/rGO nanocomposites electrode at a current density of 200 mA g^−1^ for the first 50 cycles. (c) Rate capabilities of SnO_2_/Co_3_O_4_, SnO_2_/rGO, SnO_2_/Co_3_O_4_/rGO nanocomposites. (d) Cycling performance of three types of nanocomposites electrodes, and CE of SnO_2_/Co_3_O_4_/rGO electrode.

The electrochemical performance of the nanocomposites was evaluated by integrating into a half-cell battery testing configuration composed of the synthesized active material as the working electrode and a lithium foil as the counter electrode separated by a membrane. Figure 3a shows cyclic voltammetry (CV) curves of the first three cycles of SnO_2_/Co_3_O_4_/rGO electrode within the potential range of 0.01–3 V vs Li/Li^+^. In the first cathodic cycle, there are two peaks centered at 0.65 and 0.07 V. The peak at 0.65 V corresponds to the conversion reaction of SnO_2_ and Co_3_O_4_ with Li^+^ ions into Sn and Co nanoparticles embedded in Li_2_O, and formation of solid-electrolyte interface (SEI) gel-like film at the interface of electrolyte and electrode, as it is no longer present in subsequent cycles^45^. The formation of SEI film is commonly observed in the initial cycle of anode materials^16^,^17^,^46^,^47^,^48^,^49^. The peak located at 0.07 V originates from the alloying process of Li with Sn to form Li-Sn alloy, as shown by equation 2^12^,^50^,^51^. We also have noted that the peak located at 0.89 V in the first cycle of CV curves of SnO_2_/rGO electrode (see Supporting Information, Figure S5a) is shifted slightly compared to that of SnO_2_/Co_3_O_4_/rGO electrode. It is generally accepted that this peak is related to the conversion reaction of SnO_2_ with Li ions into Sn metal nanoparticles and the observed shift is due to the introduction of Co_3_O_4_. This shift is more noticeable in the first cycle of CV curves of SnO_2_/Co_3_O_4_ electrode (see Supporting Information, Figure S6a). In the first anodic cycle, the peak at 0.56 V can be attributed to the dealloying process to form Sn and Li; and the peaks at 1.28 and 2.17 V might be due to the partial conversion reaction of Sn into SnO_2_ and conversion reaction of Co into Co_3_O_4_ with the decomposition of Li_2_O^7^,^14^,^52^. In the following cathodic cycle, the peaks located at 1 and 0.1 V are ascribed to the conversion reaction of SnO_2_ into Sn and Co_3_O_4_ into Co metal nanoparticles, and the process of Sn alloyed with lithium, respectively.

The galvanostatic discharging/charging curves of the first 50 cycles of SnO_2_/Co_3_O_4_/rGO electrode are shown in the Figure 3b. The SnO_2_/Co_3_O_4_/rGO electrode deliver capacities of 1725 and 1152 mAh g^−1^ for the 1^st^ discharge and charge cycle, with an initial CE about 66.8%. The capacity loss of 33.2% during the first cycle is generally attributed to the irreversible formation of the SEI layer on the surface of the nanocomposites and a small portion of irreversible conversion reaction of SnO_2_ into Sn during the first discharge process. The CE was further increased to almost 100% for subsequent cycles, indicating the excellent reversibility of the nanocomposites electrode. There are two plateaus located at 1.0–0.5 and 0.25–0.01 V in the initial discharge curve. The first plateau can be ascribed to the conversion reaction of SnO_2_ into Sn and partial Co_3_O_4_ into Co as well as the formation of SEI film^12^,^52^. The second plateau can be attributed to alloying process of Sn with Li^+^ ions. In the following charge cycle, there are three plateaus located at 0.4–0.7, 1.2–1.6 and 2.3–2.7 V. The first one is corresponding to the dealloy process of Li_x_Sn into Li and Sn^19^,^23^. The latter two plateaus are the partial conversion of Sn into SnO_2_ and Co into Co_3_O_4_^23^,^33^. All of these peaks are in good agreement with the CV curves.

The rate capability of the three electrodes tested under various current densities is shown in Figure 3c. The SnO_2_/Co_3_O_4_/rGO electrode exhibit reversible capacities of 1038, 966, 836, 712 and 524 mAh g^−1^ at the current densities of 100, 200, 500, 1000 and 2000 mA g^−1^, respectively. Remarkably, the specific capacity was recovered to 1059 mAh g^−1^ when the current density was reduced from 2000 to 100 mA g^−1^. Although the specific capacity of SnO_2_/rGO at low current density (100 mA g^−1^) is comparable to that of SnO_2_/Co_3_O_4_/rGO electrode, the capacity of SnO_2_/Co_3_O_4_/rGO electrode at high current density (2000 mA g^−1^) is much higher than that of SnO_2_/rGO electrode, indicating the excellent rate capability of the designed SnO_2_/Co_3_O_4_/rGO electrode. The cycling performance of the three nanocomposites electrodes was conducted at a current density of 200 mA g^−1^, as shown in Figure 3d. The synthesized SnO_2_/Co_3_O_4_/rGO nanocomposites exhibit the best stability, with a relative high discharge capacity of 1023 mAh g^−1^ and charge capacity cycle retention of 106% after 100 cycles. In contrast, the SnO_2_/rGO and SnO_2_/Co_3_O_4_ nanocomposites can only deliver lower capacities of 738 and 115 mAh g^−1^ after 100 cycles, respectively. It is also noted that the specific capacity of SnO_2_/Co_3_O_4_/rGO electrode is increased slightly with the increase of cycles. However, it is decreased for the SnO_2_/rGO and SnO_2_/Co_3_O_4_ electrodes. It is worth mentioning that the initial CE of SnO_2_/Co_3_O_4_/rGO is 66.8%, which is also higher than that of SnO_2_/rGO (60.4%) and SnO_2_/Co_3_O_4_ (51.0%) electrodes as shown in Figure S5c and S6c (see Supporting Information), respectively.

## Discussion

In order to further investigate the effects of rGO and additional Co_3_O_4_ on the electrochemical behaviors of the nanocomposites, electrochemical impedance spectroscopy (EIS) was performed after three and fifty cycles. Figure S7 (see Supporting Information) shows the Nyquist plots of the SnO_2_/Co_3_O_4_, SnO_2_/rGO, and SnO_2_/Co_3_O_4_/rGO electrodes, and fitting results are summarized in table S2 by the equivalent circuit model shown in the inset of Figure S7a. In the circuit, *R_s_* is the series or Ohmic resistance; R_f_ and CPE1 are the SEI layer resistance and the constant phase element (CPE), respectively; R_ct_ and CPE2 are attributed to the charge transfer resistance and related double layer capacitance, respectively; and Z_w_ is Warburg impedance related to the lithium-diffusion resistance^45^,^53^. R_ct_ of SnO_2_/Co_3_O_4_/rGO is reduced from 501.3 Ω (SnO_2_/Co_3_O_4_) to 19.19 Ω with the assistance of rGO. It is also interesting to find that R_ct_ of SnO_2_/rGO (27.34 Ω) is reduced by incorporation of Co_3_O_4_. This is may be due to the further reduction of GO functional groups during the synthesis process of Co_3_O_4_ on SnO_2_/rGO. With the increase of cycles, R_ct_ of SnO_2_/Co_3_O_4_/rGO is increased slightly from 19.19 Ω to 22.34 Ω. However, R_ct_ of SnO_2_/rGO is increased greatly from 27.34 Ω to 41.19 Ω, which is a possible reason for the capacity reduction slightly with cycles. At the same time, the resistance associated with the SEI layer R_f_ is increased from 22.47 Ω to 33.04 Ω for SnO_2_/Co_3_O_4_/rGO electrode, may be the result of an increase in the SEI layer thickness/resistance due to more inner active materials involved in the lithiation/delithiation process over cycles^54^.

The performance of the SnO_2_/Co_3_O_4_/rGO is one of the best those previously reported SnO_2_ based anode materials, in terms of specific capacity, initial CE and cycle stability. The reversible specific capacity of SnO_2_/Co_3_O_4_/rGO (1038 mAh g^−1^ @ 100 mA g^−1^) is higher than that of SnO_2_/GNS (810 mAh g^−1^)^13^, SnO_2_:Fe_2_O_3_:rGO (958 mAh g^−1^ @ 395 mA g^−1^)^24^, and comparable to SnO_2_/rGO (1027 mAh g^−1^ @ 100 mA g^−1^)^54^. The initial CE (66.8%) is higher than that of SnO_2_/rGO (~63.6%)^54^, SnO_2_:Fe_2_O_3_:rGO (62.4%)^24^, carbon encapsulated SnO_2_ nanoparticles (57.1%)^14^, SnO_2_ nanoboxes (46.4%)^22^, and SnO_2_ nanowires (46.9%)^50^. And SnO_2_/Co_3_O_4_/rGO nanocomposites have the highest cycle retention (106% @ 100 cycles based on charge capacity) compared to that of SnO_2_ nanorod (81.9% after 20 cycles)^55^, nanorod array (57.5% after 100 cycles)^56^, SnO_2_/GNS (70% after 30 cycles)^13^. It is generally accepted that the electrochemical performance of SnO_2_ nanocomposites can be improved by incorporation of carbonaceous materials (e.g. rGO, CNT, amorphous carbon coating) due to the increased conductivity, reduced volume change during the lithiation and delithiation process, improved adhesion with the current collector, and the prevention of aggregation of metal oxide nanoparticles^13^,^21^,^57^,^58^,^59^. It was also reported that SnO_2_ performance can be improved by incorporation of metal oxide nanoparticles. Zhu et al. developed SnO_2_/Fe_2_O_3_/rGO nanocomposites with improved cycling stability and enhanced specific capacity of 958 mAh g^−1^ at a current density of 395 mA g^−1^ 24. In this work, the improved performance is mainly attributed to the effective inhibition of the aggregation of SnO_2_ nanoparticles by the metal oxide (Fe_2_O_3_) nanoparticles during the lithiation and delithiation process^24^. However, there has yet to be clear explanations and investigations to uncover the fundamental reasons behind the observed higher than theoretical capacity.

With incorporation of other metal oxides, the improved electrochemical performance can be attributed to several possible reasons: (1) Metal oxide nanoparticles effectively prevent the volume changes of SnO_2_ during the lithiation and delithation process as the lithiation/delithiation process of the metal oxides nanoparticles does not occur at the same time; (2) these nanoparticles also prevents the aggregation of SnO_2_ nanoparticles during cycling; (3) reduced charge transfer resistance; and (4) conversion of Sn to SnO_2_ and the decomposition of Li_2_O during the delithiation process^24^. It is easy to understand the first three reasons for the improved cycling performance and rate capability. However, the increased capacity is still controversial. Although it is commonly believed that the conversion of Sn to SnO_2_ is an irreversible reaction, there are some reports which attempted to probe the presence of partial conversion reaction during delithiation process. For example, Chen et al. employed X-ray photoelectron spectroscopy (XPS) to demonstrate the disappearance of SnO_2_ when the battery was discharged to 0.01 V and exhibited again when it was charged to 3 V^26^.

**Figure 4:**
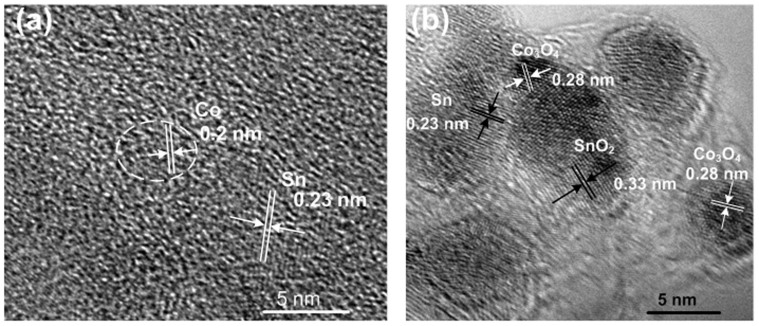
HRTEM images of SnO_2_/Co_3_O_4_/rGO electrode (a) discharged to 0.01 V and (b) charged to 3 V after 20 cycles.

The key to understand the improved electrochemical performance is the conversion reaction from Sn to SnO_2_ during the delithiation process. In order to investigate the lithiation/delithiation behavior of SnO_2_ with the effect of Co nanoparticles, two SnO_2_/Co_3_O_4_/rGO electrodes were disassembled separately. One cell was discharged to 0.01 V and another one was charged to 3 V after 20 cycles. The *ex-situ* HRTEM images of the electrodes are shown in Figure 4. As shown in Figure 4a, Co and Sn nanoparticles are found when the cell discharged to 0.01 V (lithiation process). When the cell is charged to 3.0 V (delithiation process), Co_3_O_4_ and SnO_2_ nanoparticles are clearly observed in Figure 4b. In other words, SnO_2_ nanoparticles is found after battery charging to 3 V, demonstrating the existence of SnO_2_ converted from Sn. This implies the reversible (backwards) reaction of equation (1). In addition, α-Sn is also found in the delithiation process, this phenomenon was also reported in SnO_2_/C study^14^.

**Figure 5:**
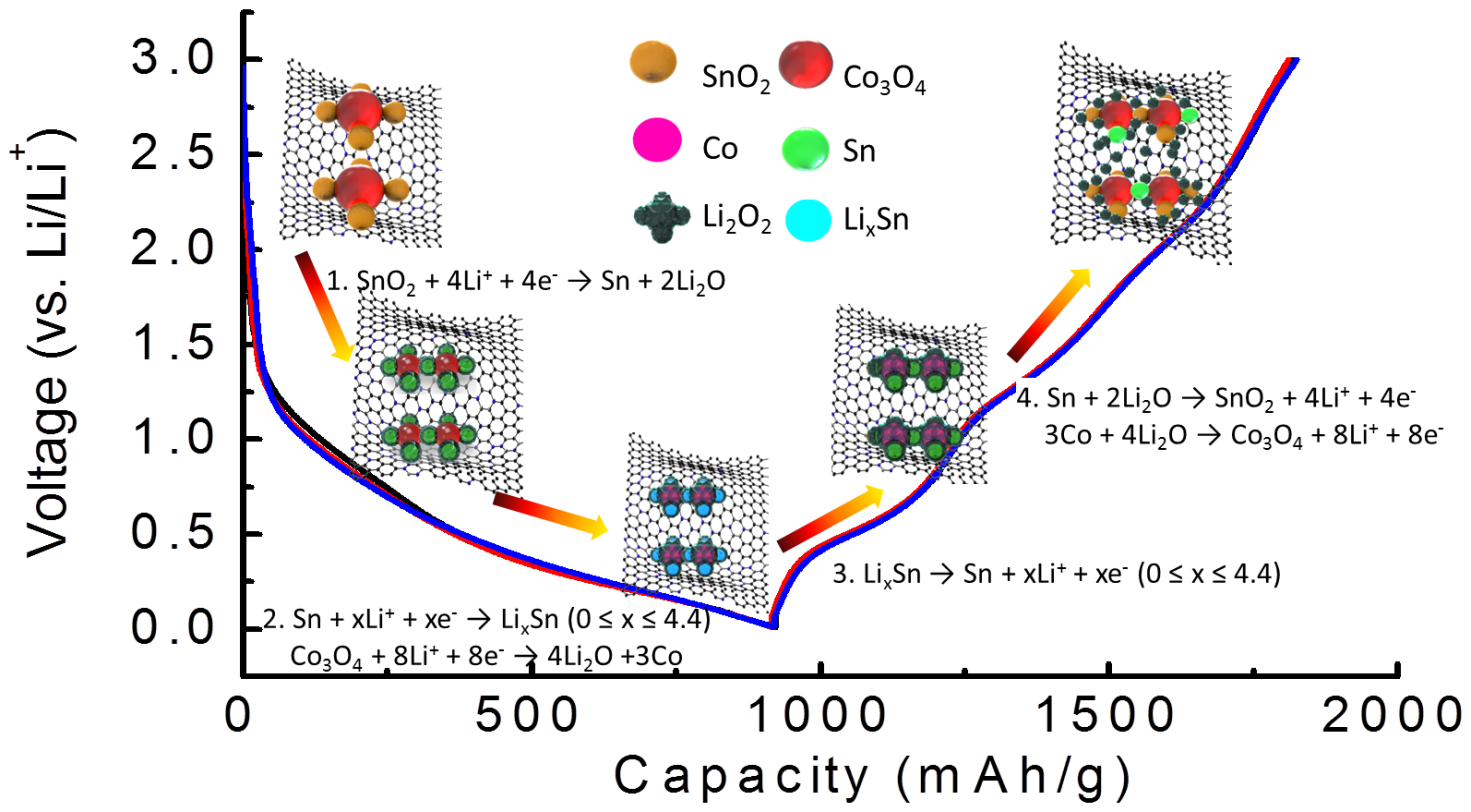
Schematic diagram of the catalytic mechanism of Co nanoparticles during the lithiation and delithiation process of SnO_2_/Co_3_O_4_/rGO nanocomposites electrode. Nano-sized Co particles promote the conversion reaction from Sn to SnO_2_ nanoparticles and the decomposition of Li_2_O during the delithiation process.

**Figure 6:**
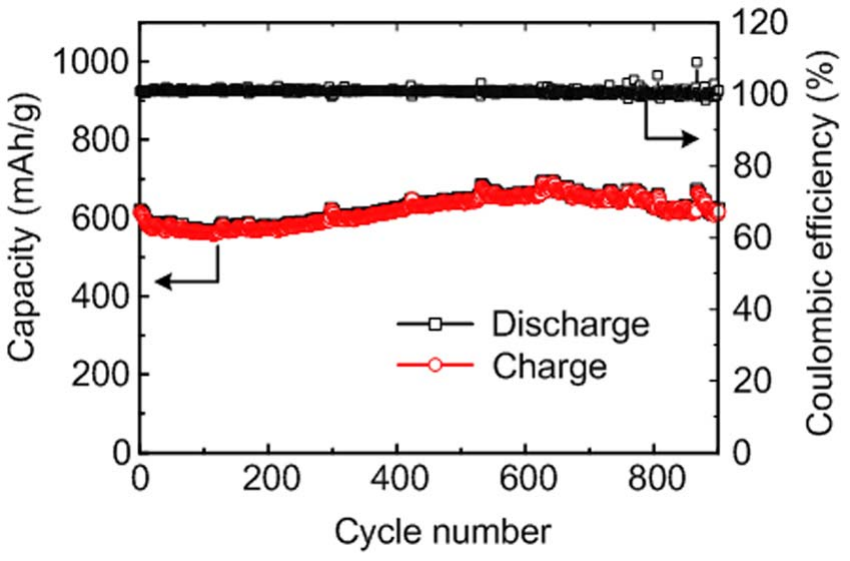
Cycling performance of SnO_2_/Co_3_O_4_/rGO electrode at a large current density of 1000 mA g^−1^. The electrode exhibits super-stable cycling performance even after 900 cycles with a nearly 100% CE, indicating excellent reversibility. The electrode was measured after 10 low current density activation process cycles.

The whole reaction mechanism of SnO_2_/Co_3_O_4_/rGO electrode lithiation/delithiation cycle can be explained in Figure 5. During the 1^st^ lithiation process (step 1), SnO_2_ nanoparticles are converted into Sn covered with Li_2_O matrix (equation 1). With the lithiation process (step 2), Sn nanoparticles are reacted with Li ions to form Li_x_Sn alloy (0 ≤ x ≤ 4.4) (equation 3), and Co_3_O_4_ nanoparticles are converted into Co nanoparticles until the end of lithiation process (equation 4 and Figure 4a)^60^. It is worth mentioning that Sn nanoparticles are also observed when the cell is discharged to 0.01 V, indicating an incomplete lithiation process. Sn nanoparticles are also found in the Sn nanowire lithiation process^61^. The absence of Li_x_Sn nanoparticles in the HRTEM images may be due to the non-crystallinity nature/amorphous phase of Li_x_Sn alloy without distinguished lattices^61^,^62^. During the lithiation process, the SEI film is formed at the interface of nanoparticles/alloy-compound and electrolyte. It is worth mentioning that the formation of SEI film guarantees the cycle stability of the electrode^63^. All particles are still held by the rGO sheet through the tight van der Waals force and chemical bonds^31^. In the delithiation process (step 3), when the cell potential is increased to 1 V, the alloyed Li_x_Sn is decomposed into Li ions and Sn metal nanoparticles (equation 5). When the potential is higher than 1 V, Co nanoparticles are began to convert into Co_3_O_4_ nanoparticles with the decomposition of Li_2_O^64^. During this stage, the decomposed Li_2_O partially oxidizes Sn into SnO_2_. With the process of delithiation (step 4), more Co nanoparticles promote the decomposition of Li_2_O (equation 6) and more Sn is converted into SnO_2_ (equation 7). As a result, the conversion of Sn to SnO_2_ is a partially reversible reaction with the assistance of Co nanoparticles during the delithiation process. In other words, Co nanoparticle acts as a catalyst to promote the conversion of Sn to SnO_2_ with the decomposition of Li_2_O.
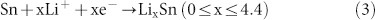

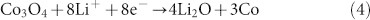

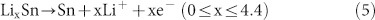

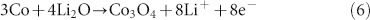

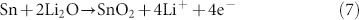
The lithium storage is determined by several factors, such as theoretical capacity of active material, specific charge storage mechanism, surface kinetics of electrochemically active materials, and transport of lithium ions and electrons at the electrode and electrolyte interface^45^,^65^. It is always a target to design new material system to achieve high electrochemical performance, including large specific capacity, high power density and energy density, as well as long cycle stability. The designed SnO_2_/Co_3_O_4_/rGO hybrid nanostructure was tested under a large current density (1000 mA g^−1^), and the result is shown in Figure 6. The SnO_2_/Co_3_O_4_/rGO electrode still can deliver a capacity of 614 mAh g^−1^ at a large current density of 1000 mA g^−1^ even after 900 cycles with a specific capacity retention of 99%. The extraordinary cycle stability at large current density can be attributed the catalytic effect promoted outstanding electrochemical performance. In addition, the specific capacity for SnO_2_/Co_3_O_4_/rGO nanocomposites is increased slightly after several cycles as shown in Figure 3d and Figure 6. The increased specific capacity may be due to three reasons: (i) more active materials activated during the cycles as the electrolyte does not fully penetrate the deeper regions of active materials in the initial several cycles as the discharge current is not small enough for fully lithiation^54^; (ii) more available active sites for lithium ion reaction due to the volume change of the SnO_2_/Co_3_O_4_/rGO^66^; and (iii) more Sn converted into SnO_2_ during the delithiation process with the assistance of Co catalyst. The application of catalyst in the field of anode material of LIBs is therefore believed to bring innovation of this area.

In summary, SnO_2_/Co_3_O_4_/rGO nanocomposites were synthesized via a simple, two-step hydrothermal/solvethermal method and employed as anode materials for LIBs. The synthesized SnO_2_/Co_3_O_4_/rGO nanocomposites exhibit excellent electrochemical performance, in terms of high specific capacity, good rate capability and long cycle stability. The improved performance is attributed to the catalytic effect of Co to promote the decomposition of Li_2_O and the conversion of Sn to SnO_2_ during the delithiation process. The catalytic role of Co is investigated by *ex-situ* TEM study. The addition Co catalyst explores a way to make SnO_2_ alloying-dealloying type anode material beyond its theoretical specific capacity and improve the rate capability and cycling performance at large current density. The demonstrated idea and synthesized nanostructure may open up a new route to develop high capacity, large power density and long cycling life LIBs for the future energy storage devices.

## Methods

### Materials synthesis

Graphene oxide (GO) was synthesized via a modified Hummer's method^67^. The SnO_2_/Co_3_O_4_/rGO nanocomposites were synthesized by two steps. For the first step of synthesis of SnO_2_/rGO, 303 mg SnCl_2_ and 6.6 mg NH_4_H_2_PO_4_ was added into the as-prepared 60 ml GO solution (1 mg/ml) with sonication for about 30 mins and continuous magnetic stirring at 50°C for 5 h. Then the mixture was transferred to a 100 mL Teflon-lined stainless steel autoclave and heated in a normal lab oven at 220°C for 24 h. After cooling down naturally, black precipitate was collected by centrifugation and washed with deionized (DI) water and ethanol for several times to remove the unreacted chemicals and residue, followed by freeze drying in a freeze dryer with vacuum at −70°C for 48 h. The collected black powder is named as SnO_2_/rGO. After synthesis of SnO_2_/rGO nanocomposites, Co_3_O_4_ nanoparticles were incorporated into SnO_2_/rGO nanocomposites by a solvothermal method^32^. In detail, 1.44 ml Co(CH_3_COO)_2_ (0.1 mol/l) and 0.8 ml NH_4_OH solution were added into 48 ml ethanol with 100 mg SnO_2_/rGO powder prepared in advanced. The precursor was heated at 80°C with continuous magnetic stirring for 10 hours, followed by transferred the mixture into a 100 ml Teflon-lined stainless steel autoclave for hydrothermal growth at 150°C for 2 hours. The final powder collection procedure was same as that of SnO_2_/rGO nanocomposites. For the control samples, SnO_2_/Co_3_O_4_ nanocomposites were prepared by the same method without the addition of GO.

### Materials characterization

The morphologies and structures of the nanocomposites were checked by field-emission scanning electron microscopy (FESEM, JSM-7600) and transmission electron microscopy (TEM, JEM-2100F). Crystal structure of the synthesized nanocomposites were performed by X-ray diffraction (XRD, Bruker D8) with Cu Ka (λ = 0.154 nm) radiation under the accelerating voltage of 40 kV. Raman spectra were carried out by a confocal Raman system with the 532 nm laser excitation (WITec Instruments Corp, Germany). Specific surface area testing was performed by N_2_ physisorption at 77 K using the Brunauer–Emmett–Teller (BET, TriStar II 3020, Micromeritics) method. The content of metal oxide in the nanocomposites was measured by thermogravimetric analysis (TGA, Shimadzu, DTG-60).

### Battery assemble process and Electrochemical measurements

The electrode slurry was prepared by mixing 80 wt% active material (SnO_2_/rGO, SnO_2_/Co_3_O_4_/rGO and SnO_2_/Co_3_O_4_ nanocomposites), 10 wt% conductive carbon black and 10 wt% polyvinyldifluoride (PVDF) binder with several drops of N-methylpyrrolidone (NMP) in a mortar agate. The slurry was then painted onto nickel foam, followed by dried overnight in a vacuum oven at 120°C and pressed into a thin slice for electrode preparation. The prepared electrode was assembled into a standard CR2032 button cell with lithium foil as counter electrode and Celgard 2400 membrane as a separator and filled with 1 M LiPF6 solution dissolved in a mixture of ethylene-carbonate–ethyl-methyl-carbonate (EC–DMC, 1:1) as the electrolyte in an argon-filled glove box. Electrochemical measurements were performed after 24 hours of the battery assembly. The cyclic voltammetry (CV) and electrochemical impedance spectroscopy (EIS) measurements were tested by an electrochemical workstation (VMP3, Bio-logic, France). The galvanostatic charge/discharge test was carried out in the voltage window of 0.01–3 V at various current densities ranging from 100 to 2000 mA g^−1^ using a battery analyzer (Neware, Shenzhen, China). The capacity of all nanocomposites is measured and calculated based on the whole mass of the nanocomposites.

In order to investigate morphology changes and detailed electrochemical behavior of the prepared SnO_2_/Co_3_O_4_/rGO nanocomposites at various lithiation/delithiation stages, two coin cells were cycled at a current density of 200 mA g^−1^ for 20 cycles and then stopped one cell at charged voltage of 3.0 V and another one at discharged voltage of 0.01 V. After that, the coin cells were disassembled in the Ar filled glove box. The electrodes were washed by NMP for several times, followed by dispersing the active material into ethanol in a small vial. After that, the active material was dropped onto a TEM sample grid for further TEM characterization. The whole process was finished in the glove box to avoid the oxidization of the sample.

## Author Contributions

Y.W. and H.Y.Y. designed experiment and analyzed data. Y.W., Z.X.H. and J.I.W. carried out the material synthesis and characterization. Y.W., Y.S. and M.D. performed the electrochemical measurement. H.Y.Y. supervised the project. All authors contributed to the writing and editing.

## Supplementary Material

Supplementary Information Supplementary Information

## References

[b1] Poizot P., Laruelle S., Grugeon S., Dupont L. & Tarascon J. M. Nano-sized transition-metaloxides as negative-electrode materials for lithium-ion batteries. Nature 407, 496–499 (2000). 11028997 10.1038/35035045

[b2] Tarascon J. M. & Armand M. Issues and challenges facing rechargeable lithium batteries. Nature 414, 359–367 (2001). 11713543 10.1038/35104644

[b3] Aricò A. S., Bruce P., Scrosati B., Tarascon J.-M. & Van Schalkwijk W. Nanostructured materials for advanced energy conversion and storage devices. Nat. Mater. 4, 366–377 (2005). 15867920 10.1038/nmat1368

[b4] Cabana J., Monconduit L., Larcher D. & Palacin M. R. Beyond Intercalation-Based Li-Ion Batteries: The State of the Art and Challenges of Electrode Materials Reacting Through Conversion Reactions. Adv. Mater. 22, E170–E192 (2010). 20730811 10.1002/adma.201000717

[b5] Liu C., Li F., Ma L. P. & Cheng H. M. Advanced Materials for Energy Storage. Adv. Mater. 22, E28–E62 (2010). 20217798 10.1002/adma.200903328

[b6] Reddy M., Subba Rao G. & Chowdari B. Metal Oxides and Oxysalts as Anode Materials for Li Ion Batteries. Chem. Rev. 113, 5364–5457 (2013). 23548181 10.1021/cr3001884

[b7] Wang Y., Xia H., Lu L. & Lin J. Excellent Performance in Lithium-Ion Battery Anodes: Rational Synthesis of Co(CO_3_)_0.5_(OH)0.11H_2_O Nanobelt Array and Its Conversion into Mesoporous and Single-Crystal Co_3_O_4_. ACS Nano 4, 1425–1432 (2010). 20146455 10.1021/nn9012675

[b8] Han S., Jang B., Kim T., Oh S. M. & Hyeon T. Simple Synthesis of Hollow Tin Dioxide Microspheres and Their Application to Lithium-Ion Battery Anodes. Adv. Funct. Mater. 15, 1845–1850 (2005).

[b9] Tang L. *et al.* Preparation, structure, and electrochemical properties of reduced graphene sheet films. Adv. Funct. Mater. 19, 2782–2789 (2009).

[b10] Wen Z., Wang Q., Zhang Q. & Li J. In Situ Growth of Mesoporous SnO_2_ on Multiwalled Carbon Nanotubes: A Novel Composite with Porous-Tube Structure as Anode for Lithium Batteries. Adv. Funct. Mater. 17, 2772–2778 (2007).

[b11] Lou X. W., Wang Y., Yuan C., Lee J. Y. & Archer L. A. Template-Free Synthesis of SnO_2_ Hollow Nanostructures with High Lithium Storage Capacity. Adv. Mater. 18, 2325–2329 (2006).

[b12] Zhou X., Dai Z., Liu S., Bao J. & Guo Y.-G. Ultra-Uniform SnO*_x_*/Carbon Nanohybrids toward Advanced Lithium-Ion Battery Anodes. Adv. Mater. 26, 3943–3949 (2014). 24664966 10.1002/adma.201400173

[b13] Paek S.-M., Yoo E. & Honma I. Enhanced cyclic performance and lithium storage capacity of SnO_2_/graphene nanoporous electrodes with three-dimensionally delaminated flexible structure. Nano Lett. 9, 72–75 (2008). 19090687 10.1021/nl802484w

[b14] Ding L. *et al.* Ultrasmall SnO_2_ Nanocrystals: Hot-bubbling Synthesis, Encapsulation in Carbon Layers and Applications in High Capacity Li-Ion Storage. Sci. Rep. 4, 4647; 10.1038/srep04647 (2014). 24732294 PMC3986698

[b15] Chen J. S. & Lou X. W. D. SnO_2_-Based Nanomaterials: Synthesis and Application in Lithium-Ion Batteries. Small 9, 1877–1893 (2013). 23386368 10.1002/smll.201202601

[b16] Liu J., Xia H., Lu L. & Xue D. Anisotropic Co_3_O_4_ porous nanocapsules toward high-capacity Li-ion batteries. J. Mater. Chem. 20, 1506–1510 (2010).

[b17] Liu J., Xia H., Xue D. & Lu L. Double-shelled nanocapsules of V_2_O_5_-based composites as high-performance anode and cathode materials for Li ion batteries. J. Am. Chem. Soc. 131, 12086–12087 (2009). 19705911 10.1021/ja9053256

[b18] Xia H. *et al.* Hierarchically structured Co_3_O_4_@ Pt@ MnO_2_ nanowire arrays for high-performance supercapacitors. Sci. Rep. 3, 2978; 10.1038/srep02978 (2013). 24132040 PMC3797991

[b19] Zhang L., Wu H. B., Liu B. & Lou X. W. Formation of porous SnO_2_ microboxes via selective leaching for highly reversible lithium storage. Eng. Environ. Sci. 7, 1013–1017 (2014).

[b20] Ramaprabhu S. & Vinayan B. Facile synthesis of SnO_2_ nanoparticles dispersed nitrogen doped graphene anode material for ultrahigh capacity lithium ion battery applications. J. Mater. Chem. A 1, 3865–3871 (2013).

[b21] Zhang N., Zhao Q., Han X., Yang J. & Chen J. Pitaya-like Sn@C nanocomposites as high-rate and long-life anode for lithium-ion batteries. Nanoscale 6, 2827–2832 (2014). 24468961 10.1039/c3nr05523j

[b22] Wang Z., Luan D., Boey F. Y. C. & Lou X. W. Fast formation of SnO_2_ nanoboxes with enhanced lithium storage capability. J. Am. Chem. Soc. 133, 4738–4741 (2011). 21401090 10.1021/ja2004329

[b23] Lian P. C. *et al.* High reversible capacity of SnO_2_/graphene nanocomposite as an anode material for lithium-ion batteries. Electrochim. Acta 56, 4532–4539 (2011).

[b24] Zhu J. *et al.* Synergetic approach to achieve enhanced lithium ion storage performance in ternary phased SnO_2_–Fe_2_O_3_/rGO composite nanostructures. J. Mater. Chem. 21, 12770–12776 (2011).

[b25] Kim W.-S. *et al.* SnO_2_@Co_3_O_4_ Hollow Nano-spheres for a Li-ion Battery Anode with Extraordinary Performance. Nano Research 7, 1128–1136 (2014).

[b26] Chen Z. *et al.* In Situ Generation of Few-Layer Graphene Coatings on SnO_2_-SiC Core-Shell Nanoparticles for High-Performance Lithium-Ion Storage. Adv. Energy Mater. 2, 95–102 (2012).

[b27] Kim C. H., Jung Y. S., Lee K. T., Ku J. H. & Oh S. M. The role of in situ generated nano-sized metal particles on the coulombic efficiency of MGeO_3_ (M = Cu, Fe, and Co) electrodes. Electrochim. Acta 54, 4371–4377 (2009).

[b28] Seng K. H., Park M.-h., Guo Z. P., Liu H. K. & Cho J. Catalytic Role of Ge in Highly Reversible GeO_2_/Ge/C Nanocomposite Anode Material for Lithium Batteries. Nano Lett. 13, 1230–1236 (2013). 23379626 10.1021/nl304716e

[b29] Liang J. *et al.* One-step in situ synthesis of SnO_2_/graphene nanocomposites and its application as an anode material for Li-ion batteries. ACS Appl. Mater. Interfaces 4, 454–459 (2012). 22188280 10.1021/am201541s

[b30] Cao A. *et al.* A Facile One-step Method to Produce Graphene–CdS Quantum Dot Nanocomposites as Promising Optoelectronic Materials. Adv. Mater. 22, 103–106 (2010). 20217706 10.1002/adma.200901920

[b31] Wu Z.-S. *et al.* Graphene/metal oxide composite electrode materials for energy storage. Nano Energy 1, 107–131 (2012).

[b32] Liang Y. Y. *et al.* Co_3_O_4_ nanocrystals on graphene as a synergistic catalyst for oxygen reduction reaction. Nat. Mater. 10, 780–786 (2011). 21822263 10.1038/nmat3087

[b33] Wang Y. *et al.* Onion-like carbon matrix supported Co_3_O_4_ nanocomposites: a highly reversible anode material for lithium ion batteries with excellent cycling stability. J. Mater. Chem. A. 1, 5212–5216 (2013).

[b34] Yao M. *et al.* Wet chemical synthesis and magnetic properties of core–shell nanocolumns of Ni(OH)_2_@Co(OH)_2_ and their oxides. CrystEngComm 13, 2593–2598 (2011).

[b35] Li B. J. *et al.* Co_3_O_4_@graphene Composites as Anode Materials for High-Performance Lithium Ion Batteries. Inorg. Chem. 50, 1628–1632 (2011). 21244033 10.1021/ic1023086

[b36] Dieguez A., Romano-Rodrıguez A., Vila A. & Morante J. The complete Raman spectrum of nanometric SnO_2_ particles. J. Appl. Phys. 90, 1550–1557 (2001).

[b37] Ferrari A. *et al.* Raman spectrum of graphene and graphene layers. Phys. Rev. Lett. 97, 187401 (2006). 17155573 10.1103/PhysRevLett.97.187401

[b38] Eigler S. & Hirsch A. Chemistry with Graphene and Graphene Oxide—Challenges for Synthetic Chemists. Angew. Chem. Int. Ed. 53, 7720–7738 (2014). 10.1002/anie.201402780 24962439

[b39] Dreyer D. R., Park S., Bielawski C. W. & Ruoff R. S. The chemistry of graphene oxide. Chem. Soc. Rev. 39, 228–240 (2010). 20023850 10.1039/b917103g

[b40] Ferrari A. C. Raman spectroscopy of graphene and graphite: disorder, electron–phonon coupling, doping and nonadiabatic effects. Solid State Commun. 143, 47–57 (2007).

[b41] Wang Y., Yu S. F., Sun C. Y., Zhu T. J. & Yang H. Y. MnO_2_/onion-like carbon nanocomposites for pseudocapacitors. J. Mater. Chem. 22, 17584–17588 (2012).

[b42] Wang Y. *et al.* Pre-lithiation of onion-like carbon/MoS_2_ nano-urchin anodes for high-performance rechargeable lithium ion batteries. Nanoscale 6, 8884–8890 (2014). 24962690 10.1039/c4nr01553c

[b43] Xu C., Wang X., Zhu J., Yang X. & Lu L. Deposition of Co_3_O_4_ nanoparticles onto exfoliated graphite oxide sheets. J. Mater. Chem. 18, 5625–5629 (2008).

[b44] Li J., Tang S., Lu L. & Zeng H. C. Preparation of nanocomposites of metals, metal oxides, and carbon nanotubes via self-assembly. J. Am. Chem. Soc. 129, 9401–9409 (2007). 17616130 10.1021/ja071122v

[b45] Wang Y. *et al.* Core-leaf onion-like carbon/MnO_2_ hybrid nano-urchins for rechargeable lithium-ion batteries. Carbon 64, 230–236 (2013).

[b46] Gao X. *et al.* Novel Germanium/Polypyrrole Composite for High Power Lithium-ion Batteries. Sci. Rep. 4, 6095; 10.1038/srep06095 (2014). 25168783 PMC4148674

[b47] Hu L. *et al.* CoMn_2_O_4_ spinel hierarchical microspheres assembled with porous nanosheets as stable anodes for lithium-ion batteries. Sci. Rep. 2, 986; 10.1038/srep00986 (2012). 23248749 PMC3523291

[b48] Wang X. *et al.* TiO_2_ modified FeS nanostructures with enhanced electrochemical performance for lithium-ion batteries. Sci. Rep. 3, 2007; 10.1038/srep02007 (2013). 23774372 PMC3684812

[b49] Yan N. *et al.* Hollow Porous SiO_2_ Nanocubes Towards High-performance Anodes for Lithium-ion Batteries. Sci. Rep. 3, 1568; 10.1038/srep01568 (2013). 23535780 PMC3610094

[b50] Park M. S. *et al.* Preparation and Electrochemical Properties of SnO_2_ Nanowires for Application in Lithium-Ion Batteries. Angew. Chem. 119, 764–767 (2007). 10.1002/anie.200603309 17163569

[b51] Wang Y., Su F., Lee J. Y. & Zhao X. Crystalline carbon hollow spheres, crystalline carbon-SnO_2_ hollow spheres, and crystalline SnO_2_ hollow spheres: synthesis and performance in reversible Li-ion storage. Chem. Mater. 18, 1347–1353 (2006).

[b52] Wang Y. *et al.* Designed functional systems from peapod-like Co@ carbon to Co_3_O_4_@ carbon nanocomposites. ACS Nano 4, 4753–4761 (2010). 20666372 10.1021/nn1004183

[b53] Liu S.-Y. *et al.* Nanocrystal manganese oxide (Mn_3_O_4_, MnO) anchored on graphite nanosheet with improved electrochemical Li-storage properties. Electrochim. Acta 66, 271–278 (2012).

[b54] Li L., Kovalchuk A. & Tour J. M. SnO_2_-Reduced Graphene Oxide Nanoribbons as Anodes for Lithium Ion Batteries with Enhanced Cycling Stability. Nano Research 7, 1319–1326 (2014).

[b55] Chen S. *et al.* Kinetics-controlled growth of aligned mesocrystalline SnO_2_ nanorod arrays for lithium-ion batteries with superior rate performance. Nano Research 6, 243–252 (2013).

[b56] Liu J. *et al.* Direct growth of SnO_2_ nanorod array electrodes for lithium-ion batteries. J. Mater. Chem. 19, 1859–1864 (2009).

[b57] Kim D.-W. *et al.* Highly conductive coaxial SnO_2_-In_2_O_3_ heterostructured nanowires for Li ion battery electrodes. Nano Lett. 7, 3041–3045 (2007). 17760477 10.1021/nl0715037

[b58] Luo J. *et al.* Three-Dimensional Graphene Foam Supported Fe_3_O_4_ Lithium Battery Anodes with Long Cycle Life and High Rate Capability. Nano Lett. 13, 6136–6143 (2013). 24219630 10.1021/nl403461n

[b59] Hu Y. Y. *et al.* Origin of additional capacities in metal oxide lithium-ion battery electrodes. Nat. Mater. 12, 1130–1136 (2013). 24185759 10.1038/nmat3784

[b60] Su Q., Zhang J., Wu Y. & Du G. Revealing the electrochemical conversion mechanism of porous Co_3_O_4_ nanoplates in lithium ion battery by in situ transmission electron microscopy. Nano Energy 9, 264–272 (2014).

[b61] Huang J. Y. *et al.* In Situ Observation of the Electrochemical Lithiation of a Single SnO_2_ Nanowire Electrode. Science 330, 1515 (2010). 21148385 10.1126/science.1195628

[b62] Zhang L. Q. *et al.* Controlling the lithiation-induced strain and charging rate in nanowire electrodes by coating. ACS Nano 5, 4800–4809 (2011). 21542642 10.1021/nn200770p

[b63] Guo Y. G., Hu J. S. & Wan L. J. Nanostructured materials for electrochemical energy conversion and storage devices. Adv. Mater. 20, 2878–2887 (2008).

[b64] Wu Z.-S. *et al.* Graphene anchored with Co_3_O_4_ nanoparticles as anode of lithium ion batteries with enhanced reversible capacity and cyclic performance. ACS Nano 4, 3187–3194 (2010). 20455594 10.1021/nn100740x

[b65] Jamnik J. & Maier J. Nanocrystallinity effects in lithium battery materials Aspects of nano-ionics. Part IV. Phys. Chem. Chem. Phys. 5, 5215–5220 (2003).

[b66] Guo J. C., Liu Q., Wang C. S. & Zachariah M. R. Interdispersed Amorphous MnOx-Carbon Nanocomposites with Superior Electrochemical Performance as Lithium-Storage Material. Adv. Funct. Mater. 22, 803 (2012).

[b67] Hummers W. S. & Offeman R. E. Preparation of Graphitic Oxide. J. Am. Chem. Soc. 80, 1339–1339 (1958).

