# Supplementary material for: Designed hybrid nanostructure with catalytic effect: beyond the theoretical capacity of SnO2 anode material for lithium ion batteries
Source: Sci Rep. 2015 Mar 17;5:9164. doi: 10.1038/srep09164 (PMC4361932; doi:10.1038/srep09164)
Supplement: Supplementary Information [file srep09164-s1.pdf]

Supporting Information

SUBJECT AREAS

BATTERIES, MATERIALS FOR ENERGY AND CATALYSIS, MATERIALS SCIENCE

Correspondence and requests for materials should be addressed to H.Y. Yang(yanghuiying@sutd.edu.sg)

Designed hybrid nanostructure with catalytic effect: beyond the theoretical capacity of SnO_2 anode material for lithium ion batteries

Ye Wang, Zhi Xiang Huang, Yumeng Shi, Jen It Wong, Meng Ding, Hui Ying Yang

Pillar of Engineering Product Development, Singapore University of Technology and Design, 8 Somapah Road, 487372, Singapore

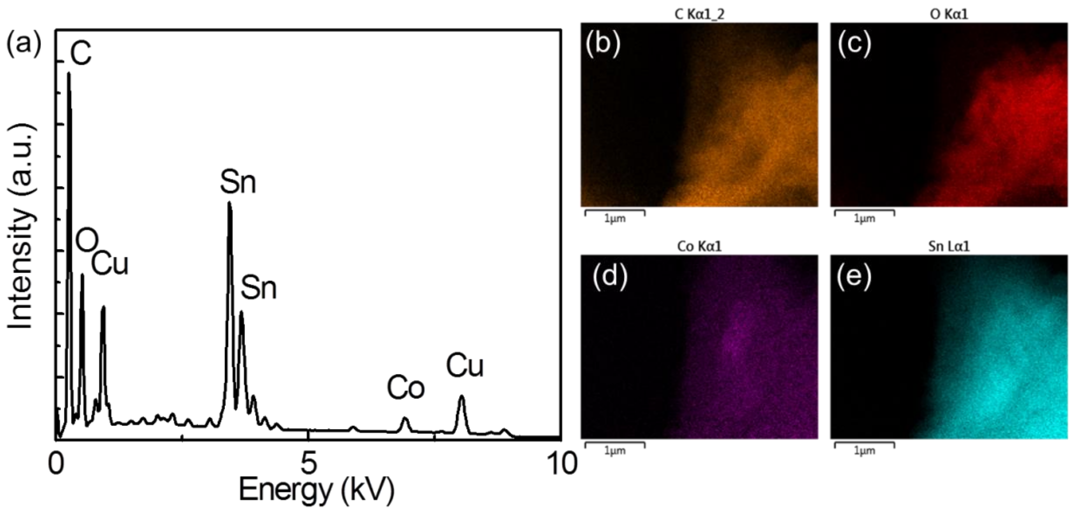

Figure S1. (a) EDX and elements distribution (b) carbon, (c) oxygen, (d) tin, (e) cobalt of SnO_2/Co_3O_4/rGO nanocomposites. The measured nanocomposite was carried out on TEM Cu grid holder, Cu peak can be found in Figure S1a.

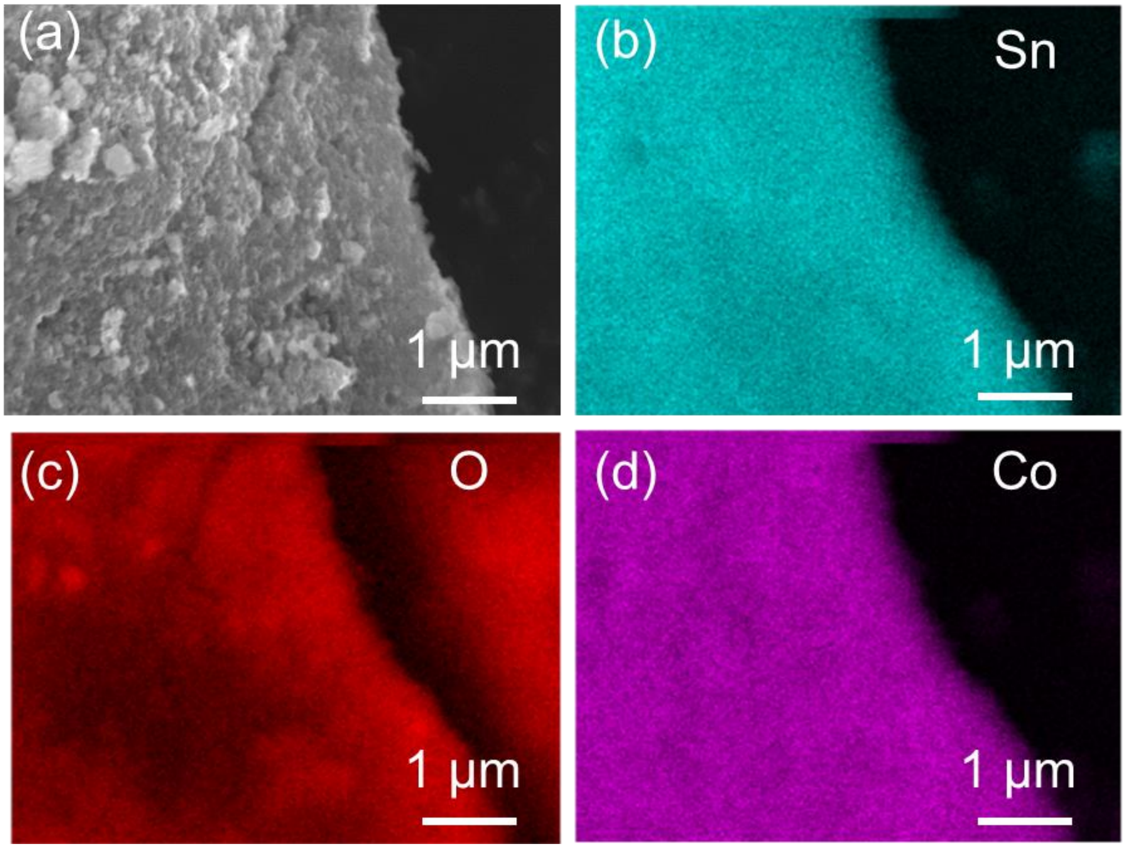

Figure S2. (a) SEM image of SnO_2/Co_3O_4 nanocomposites, and related elements distribution (b) tin, (c) oxygen and (d) cobalt.

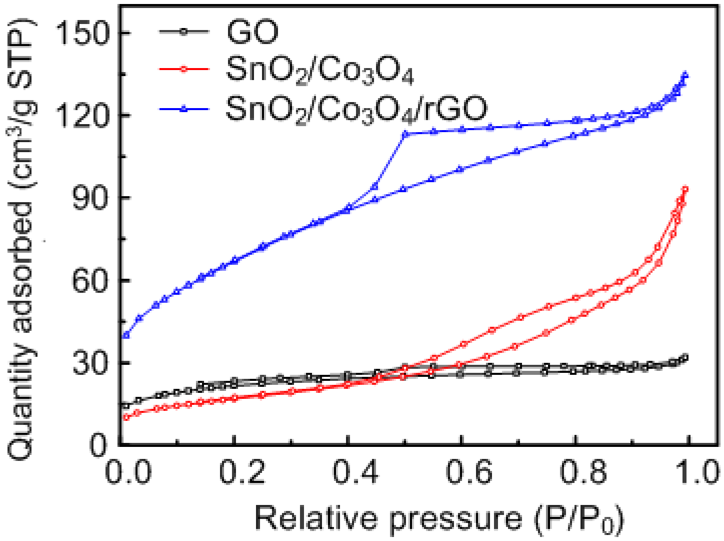

Figure S3. Nitrogen adsorption–desorption isotherms of the pure GO, SnO_2/Co_3O_4 and SnO_2/Co_3O_4/rGO nanocomposites.

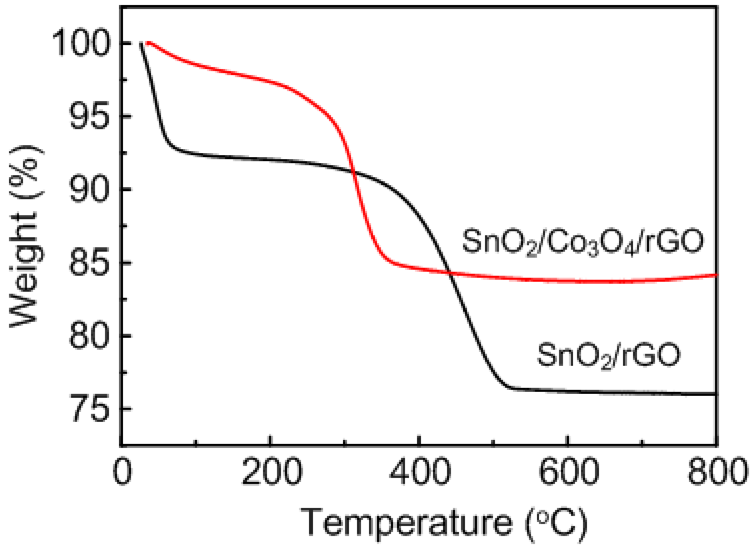

Figure S4. TGA curves of SnO_2/rGO and SnO_2/Co_3O_4/rGO nanocomposites.

Calculation of the content of Co_3O_4 in $SnO_2(Co_3O_4)_{0.174}$/rGO nanocomposites

Table S1 Content of moisture, GO and metal oxide in SnO_2/rGO, and SnO_2/Co_3O_4/rGO nanocomposites according to TGA result shown in Figure S4.

	Moisture	rGO	Metal oxide
SnO_2/rGO	8%	16%	76%
SnO_2/Co_3O_4/rGO	1.4%	15%	83.6%

The content of Co_3O_4 in the SnO_2/Co_3O_4/rGO nanocomposites is calculated based on the ratio of SnO_2 and rGO in SnO_2/Co_3O_4/rGO nanocomposites is same as that of SnO_2/rGO nanocomposites. In SnO_2/rGO nanocomposites, the ratio of rGO : SnO_2 = 16 wt% : 76 wt% = 17.4 wt% : 82.6 wt%. In SnO_2/Co_3O_4/rGO nanocomposites, the ratio of rGO : (SnO_2 + Co_3O_4) = 15 wt% : 83.6 wt% as indicated in Table S1. By expressing the content of rGO, SnO_2 and Co_3O_4 in terms of x, y and z, respectively, the following equations are derived:

$$x : y = 17.4\% : 82.6\%$$
$$y + z = 83.6\%$$
$$x = 15\%$$

Therefore, the content of Co_3O_4 and SnO_2 are calculated to be 12.4% and 71.2%, respectively. In other words, Co_3O_4 : SnO_2 = 0.174 : 1. Therefore, the SnO_2/Co_3O_4/rGO nanocomposites was expressed as $SnO_2(Co_3O_4)_{0.174}$/rGO.

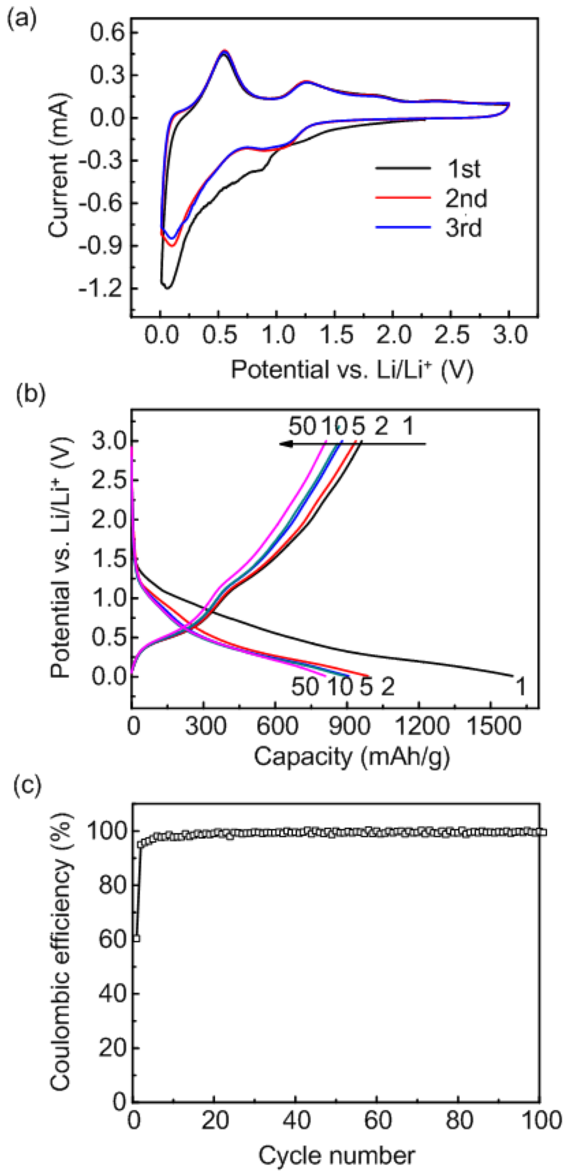

Figure S5. Electrochemical peformance SnO_2/rGO nanocomposites. (a) CV curves of SnO_2/rGO electrode of the first 3 cycles at a scan rate of 0.1 mV s^{-1} in a potential range of 0.01–3 V *vs.* Li/Li^+. (b) Galvanostatic discharge/charge curves of SnO_2/rGO electrode at a current density of 200 mA g^{-1} for first 50 cycles. (c) CE of SnO_2/rGO electrode.

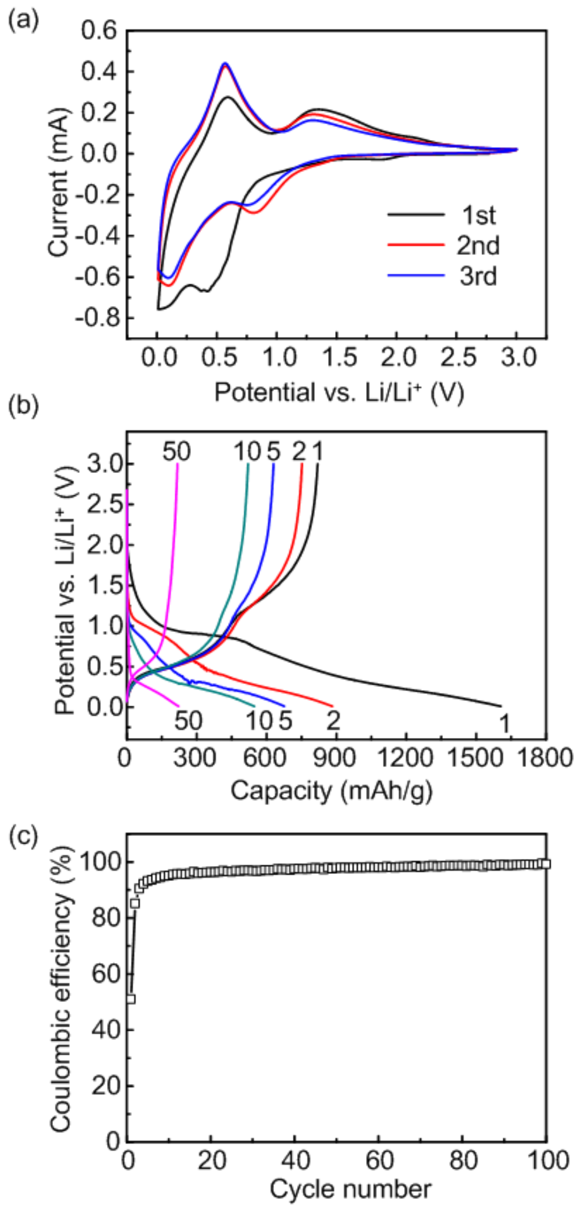

Figure S6. Electrochemical peformance SnO_2/Co_3O_4 nanocomposites. (a) CV curves of SnO_2/Co_3O_4 electrode of the first 3 cycles at a scan rate of 0.1 mV s^{-1} in a potential range of 0.01–3 V *vs.* Li/Li^+. (b) Galvanostatic discharge/charge curves of SnO_2/Co_3O_4 electrode at a current density of 200 mA g^{-1} for 50 cycles. (c) CE of SnO_2/Co_3O_4 electrode.

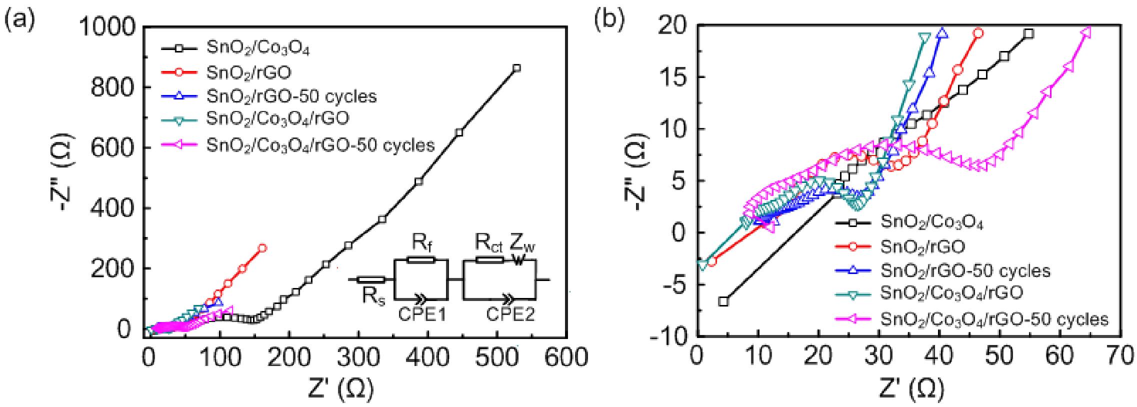

Figure S7. (a) EIS spectra and (b) an enlarged area at the high frequency range of SnO_2/Co_3O_4, SnO_2/rGO, SnO_2/Co_3O_4/rGO nanocomposite electrodes. Inset in (a) is the equivalent circuit model used for the fittings.

Table S2. Fitting results of the EIS curves in **Figure S7a** using the equivalent circuit.

Sample	Rs (Ω)	Rf (Ω)	CPE1		Rct (Ω)	CPE2		Z_W (Ω^{-1})
			Y	n		Y	n	
SnO_2/Co_3O_4	19.83	174.8	2.74×10^{-4}	0.43	501.3	1.10×10^{-3}	0.87	5.19×10^{-4}
SnO_2/rGO	10.64	30.23	5.33×10^{-4}	0.52	27.34	1.90×10^{-3}	0.89	3.64×10^{-3}
SnO_2/rGO -50 cycles	9.367	23.62	2.08×10^{-3}	0.36	41.19	1.13×10^{-3}	0.74	1.53×10^{-3}
SnO_2/Co_3O_4/rGO	7.617	22.47	8.07×10^{-4}	0.46	19.19	9.70×10^{-3}	0.89	9.57×10^{-3}
SnO_2/Co_3O_4/rGO-50 cycles	8.69	33.04	2.26×10^{-4}	0.56	22.34	8.60×10^{-3}	0.80	1.39×10^{-2}
